# Heart failure in systemic right ventricle: Mechanisms and therapeutic options

**DOI:** 10.3389/fcvm.2022.1064196

**Published:** 2023-01-10

**Authors:** Belal Nedal Sabbah, Tarek Ziad Arabi, Areez Shafqat, Saleha Abdul Rab, Adhil Razak, Dimpna Calila Albert-Brotons

**Affiliations:** ^1^College of Medicine, Alfaisal University, Riyadh, Saudi Arabia; ^2^Department of Pediatric Cardiology, Pediatric Heart Failure and Heart Transplant, Heart Center, King Faisal Specialist Hospital and Research Centre, Riyadh, Saudi Arabia

**Keywords:** systemic right ventricle, heart failure, dextro-transposition of the great arteries, congenitally corrected transposition of the great arteries, atrial switch repair, tricuspid regurgitation, congenital heart disease, pediatric cardiology

## Abstract

d-loop transposition of the great arteries (d-TGA) and congenitally corrected transposition of the great arteries (cc-TGA) feature a right ventricle attempting to sustain the systemic circulation. A systemic right ventricle (sRV) cannot support cardiac output in the long run, eventually decompensating and causing heart failure. The burden of d-TGA patients with previous atrial switch repair and cc-TGA patients with heart failure will only increase in the coming years due to the aging adult congenital heart disease population and improvements in the management of advanced heart failure. Clinical data still lags behind in developing evidence-based guidelines for risk stratification and management of sRV patients, and clinical trials for heart failure in these patients are underrepresented. Recent studies have provided foundational data for the commencement of robust clinical trials in d-TGA and cc-TGA patients. Further insights into the multifactorial nature of sRV failure can only be provided by the results of such studies. This review discusses the mechanisms of heart failure in sRV patients with biventricular circulation and how these mediators may be targeted clinically to alleviate sRV failure.

## 1. Introduction

**FIGURE 1:**
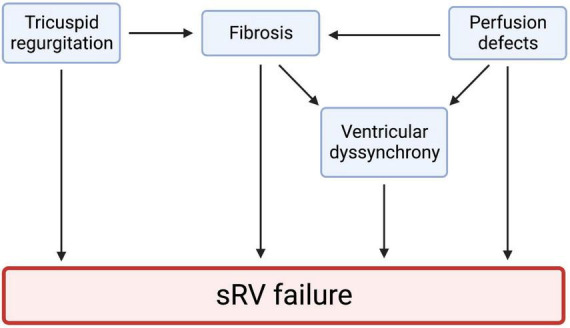
Tricuspid regurgitation, myocardial fibrosis, perfusion defects, and ventricular dyssynchrony all play a role in sRV failure. These four mechanisms may be highly interlinked. Studies have shown that ventricular dyssynchrony results from hypertrophy, ischemia, and heart failure. Cardiac fibrosis is also a significant predictor of ventricular dyssynchrony. These observations hint at a highly interlinked pathophysiology of sRV failure. We created the figure with biorender.com.

Right ventricular (RV) dysfunction is a key prognostic determinant of patient status in various cardiac diseases, including pulmonary hypertension (PHTN), cor pulmonale, left-sided heart failure, valvular heart disease, and coronary artery disease. Congenital heart diseases (CHDs) such as tetralogy of Fallot, transposition of the great vessels (TGA), Ebstein’s anomaly, and Eisenmenger syndrome are important causes of RV dysfunction (1). About 10–12% of all CHDs feature a systemic right ventricle (sRV) (2). When made to pump against the high afterload of the systemic circulation, the sRV undergoes various structural and mechanical changes to maintain cardiac output (Figure 1). These changes chronically become maladaptive, resulting in heart failure (3). d-loop TGA arteries (d-TGA) after atrial switch repair and congenitally corrected TGA (cc-TGA) are two frequently encountered CHDs that feature an sRV, with chronic pressure and volume overload expectedly causing sRV dysfunction. Furthermore, commonly associated lesions, such as ventricular septal defect, arrhythmias, tricuspid regurgitation (TR), and conduction abnormalities compound sRV dysfunction in TGA patients (4).

d-TGA features isolated ventriculoarterial discordance, resulting in venous return to the right heart being pumped back into the systemic circuit by the right ventricle and oxygenated blood returning to the left atrium (LA) being pumped back into the pulmonary circulation by the left ventricle (LV). d-TGA patients present with neonatal cyanosis and require emergent surgical correction. Left untreated, d-TGA carries a 90% mortality risk in the first year of life (5, 6). The atrial switch operation, in which systemic blood is baffled to the LV, and pulmonary venous blood to the right, restores physiologic blood circulation in d-TGA but does not anatomically correct the sRV. While this was historically the procedure of choice in the 1970s–1980s, it has now been replaced by the arterial switch operation. The arterial switch connects the aorta and pulmonary trunk to their anatomically correct ventricles, thereby offering anatomical repair and excellent clinical outcomes (7). On long-term follow-up, d-TGA patients undergoing the arterial switch have a post-operative hazard for death resembling that of the general population (8). However, the widespread use of atrial switch repair in the 1970s and 1980s, along with improvements in medical and invasive therapy for cardiac complications, has created an aging post-atrial switch d-TGA population with an sRV that is at an annually increasing risk of major cardiac complications. Only 60% of d-TGA patients treated with an atrial switch remain alive 30 years after the procedure (9). SCD is the most common cause of death in d-TGA patients after the atrial switch surgery, especially in adolescence and early adulthood (10).

In contrast, cc-TGA exhibits discordant atrioventricular and ventriculoarterial connections. The right atrium (RA) is connected to the subpulmonary LV through the mitral valve, while the LA empties blood into the sRV via the tricuspid valve. Therefore, as physiologic blood circulation is preserved, cc-TGA infants do not present with cyanosis and can be asymptomatic at birth. However, associated anomalies such as a ventricular septal defect, an Ebsteinoid malformation of the tricuspid valve, subpulmonic stenosis, accompany cc-TGA in 80% of cases and may necessitate surgery during infancy (11). In the absence of associated lesions, cc-TGA patients remain asymptomatic, presenting in adulthood with sRV dysfunction manifesting clinically as valvular disease, heart block, and heart failure. However, unlike d-TGA, heart failure in cc-TGA is not uniform in late adulthood; some elderly individuals with cc-TGA can remain completely asymptomatic. The likelihood of clinical deterioration and mortality in cc-TGA patients greatly depends on the presence of tricuspid insufficiency (11).

Therefore, the pathophysiology of sRV failure is multifactorial, and the unclear prognosis of sRV patients hinders effective management. Furthermore, clinical trials evaluating traditional heart failure drugs, such as angiotensin-converting enzyme inhibitors (ACEis), angiotensin-receptor blocker (ARBs), and others, have yielded disappointing results. Recommendations for managing arrhythmias and conduction abnormalities in TGA patients are also unclear. Lastly, only a handful of single-center data have been published regarding the role of mechanical circulatory support (MCS) devices in advanced sRV failure patients. Therefore, in this review, we summarize the primary mechanisms of sRV failure and review the latest studies on the stratification and management of sRV patients.

## 2. Mechanisms of systemic right ventricle failure

### 2.1. Myocardial fibrosis

Myocardial fibrosis is a pathological process induced by many cardiac pathologies, including those characterized by a pressure overload, such as aortic stenosis and hypertension. Myocardial fibrosis has proven predictive value in determining the risk of heart failure progression and mortality (12, 13). Detecting late-gadolinium enhancement (LGE) on cardiac magnetic resonance imaging (CMR) is the gold standard for detecting and characterizing myocardial fibrosis. Studies have also used T1 mapping and calculating extracellular volume (ECV) fraction, which correlates histologically with edema and interstitial expansion of the myocardium (14, 15). A fragmented QRS (fQRS) complex on electrocardiography (ECG) indicates inhomogeneous ventricular depolarization due to myocardial fibrosis (16).

sRV patients also experience a state of chronic pressure overload as the sRV pumps blood against the afterload of the systemic circulation, suggesting that myocardial fibrosis may be a prominent structural alteration in sRV patients. Indeed, compared with other CHDs, such as tetralogy of Fallot and cyanotic diseases, sRV patients feature the highest fibrotic indices (17). The presence of LGE varies between different cohorts from 41 to 56% (18, 19). sRV LGE is associated with reduced sRV ejection fraction and worsening clinical presentations, including tachyarrhythmia, heart failure-related hospitalization, transplantation, and death (18). Helsen et al. retrospectively studied 89 d-TGA patients post-atrial switch (median follow-up ∼16.9 years) and concluded that the presence of fQRS complexes in d-TGA patients (29%) was significantly associated with the combined primary outcome of cardiovascular mortality, MCS device implantation, or heart transplantation (20). However, the presence of fQRS was not correlated with radiological findings of sRV fibrosis/scarring, such as LGE or increased ECV.

Serum levels of collagen turnover markers have been used as indicators of myocardial fibrosis. A hallmark of fibrosis is the transformation of cardiac fibroblasts into myofibroblasts expressing a-smooth muscle antigen (α-SMA) (21). α-SMA-expressing myofibroblasts deposit collagen (22), leading to a vicious cycle of collagen turnover with an imbalance in the levels of metalloproteinase-1 (MMP-1) and tissue inhibitor of MMP-1 (TIMP-1) levels (21). In d-TGA patients after the atrial switch procedure, serum levels of procollagen type III amino-terminal propeptide (PIIINP) and collagen type I carboxy-terminal telopeptide (CITP) are significantly associated with a higher sRV mass, with the latter also associated with sRV LGE (23). Furthermore, an increased serum pro-MMP-1:TIMP-1 ratio is associated with sRV wall stress and ejection fraction, LGE on CMR, and adverse clinical outcomes on follow-up, including New York Heart Association (NYHA) functional class ≥ 3, clinically significant arrhythmias, and death (23, 24). These markers may help stratify d-TGA patients according to the risk of adverse clinical outcomes and dictate appropriate management strategies.

**FIGURE 2:**
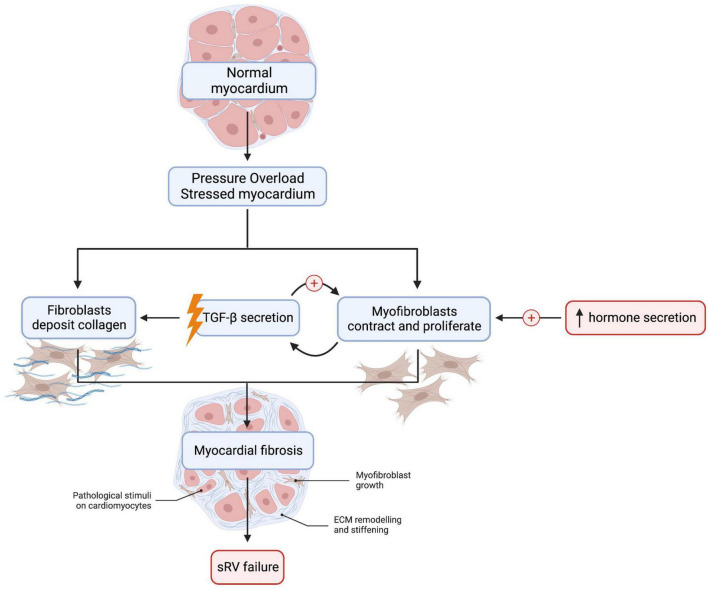
Myocardial fibrosis is detected in many sRV patients and increases the risk of adverse cardiac outcomes such as heart failure. Cardiac fibroblast and myofibroblast activation is an essential mediator of fibrosis in left ventricular fibrosis, but this remains to be demonstrated in sRV. TGF-β secretion by myofibroblasts sets up a positive feedback loop that promotes tissue fibrosis. Abnormalities in MMP:TIMP ratio may also result in dysregulated collagen turnover. Lastly, neurohormonal activation is detected in sRV patients: aldosterone, angiotensin II, epinephrine, and norepinephrine are elevated in sRV patients and stimulate collagen production in the myocardium. However, clinical trials thus far have failed to show a beneficial effect of inhibiting the sympathetic and RAAS systems in sRV patients. We created the figure with biorender.com.

From a mechanistic standpoint, RV pressure overload induces collagen deposition by fibroblasts and myofibroblast proliferation and contraction (Figure 2) (21). These changes are associated with increased expression of β1 integrin on fibroblasts, which is a receptor for transforming growth factor-β (TGF-β) (21, 25, 26). Volume overload causes TGF-β secretion by myofibroblasts, which, in turn, activates myofibroblasts in an autocrine fashion and mediates fibrosis (27). TGF-β pathways may therefore be beneficial as a therapeutic target. For example, treatment with allicin—derived from garlic—prevented RV fibrosis in rat models of pulmonary arterial hypertension (28). Neurohormones, such as aldosterone, angiotensin II, epinephrine, and norepinephrine, also activate cardiac myofibroblasts and stimulate collagen production (29, 30).

Other data argue against a significant role of myocardial fibrosis in sRV failure. For example, Fratz et al. found no myocardial fibrosis or scarring, assessed by LGE and positron emission tomography (PET) scan, respectively, in 18 d-TGA patients over 20 years after the atrial switch operation, even in patients with reduced sRV ejection fraction and wall motion abnormalities (31). Similarly, no myocardial fibrosis or scarring was detected in cc-TGA patients with reduced sRV ejection fraction or wall motion abnormalities (31). Priem et al. tested the hypothesis that myocardial fibrosis underpins sRV systolic dysfunction in 20 patients (32). The study found myocardial scarring in only one patient, while 90% of patients had NYHA class I or II sRV failure, 95% had TR, and 65% arrhythmias, indicating that myocardial fibrosis and scarring do not play causal roles in sRV dysfunction and complications (32).

Cheung et al. utilized T1 mapping on CMR to assess sRV and subpulmonary LV fibrosis in d-TGA patients after the atrial switch operation and found the LV to be significantly more fibrotic than the sRV, but neither correlated with the ejection fractions of the respective ventricles (33). Another recent study assessing 13 adult sRV patients (8 d-TGA patients after the atrial switch and 5 cc-TGA patients) also showed that fibrosis—assessed by ECV fraction—disproportionately involved the LV. Differences in RV ECV between sRV patients and healthy controls were not statistically significant, whereas an elevated LV ECV correlated significantly with a lower LV ejection fraction (34). Indeed, a study investigating subpulmonary LV and sRV dysfunction in 157 sRV patients (89 d-TGA and 68 cc-TGA) found that subpulmonary LV systolic dysfunction assessed by echocardiography was strongly predictive of NYHA class 3–4 heart failure (35). These findings suggest that myocardial fibrosis differentially involves the LV, which reduces LV ejection fraction and contributes to heart failure in sRV patients, whereas sRV fibrosis may not play a major role in precipitating sRV failure. sRV fibrosis and scarring may not play major roles in precipitating sRV failure. However, the prognostic importance of monitoring LV fibrosis as part of monitoring disease progression remains investigational.

### 2.2. Myocardial ischemia and perfusion defects

Myocardial ischemia and perfusion defects are crucial players in heart failure with preserved (HFpEF) and reduced ejection fractions (HFrEF) (36). For instance, reduced coronary flow reserve (CFR) is independently associated with diastolic dysfunction, conferring a fivefold increased risk of HFpEF-related hospitalizations (37). Impaired CFR in cc-TGA and d-TGA post-atrial switch patients has been detected on PET scans (38, 39). In one study, 33% of d-TGA patients had moderate-to-severe perfusion abnormalities at rest. Another 20% demonstrated reduced CFR upon exercise, which correlated positively with worse sRV and subpulmonic LV function (38). Another study of 5 cc-TGA patients revealed fixed perfusion defects in the entire cohort and reversible myocardial ischemia in 4 patients (40).

These findings indicate that monitoring for ischemic myocardial damage may identify sRV patients at risk of adverse effects, including heart failure. High-sensitivity Troponin-T (hsTnT) is a diagnostic marker for acute coronary syndrome (ACS). It has also been used as a biomarker for cardiac dysfunction in adult CHDs. Profound hsTnT elevations above the 99th percentile are detected in sRV patients and CHDs with PHTN (41). hsTnT levels also correlate with higher degrees of systolic dysfunction, non-sinus rhythm, a higher NYHA heart failure classification, and elevated NT-proBNP levels (41). Kowalik et al. associated higher serum hsTnT levels with worse echocardiographic parameters of cardiac function and higher NYHA functional class, but, due to the cross-sectional nature of this study, a temporal relationship in which hsTnT elevations precede cardiac dysfunction could not be established (42). The same authors later conducted a prospective study and reported that hsTnT levels were the best predictor of a composite clinical outcome of worsening of heart failure, vascular events, TR, clinically relevant arrhythmias, MCS device implantation, heart transplant, and death (HR = 6.25, *P* = 0.02) (43).

**FIGURE 3:**
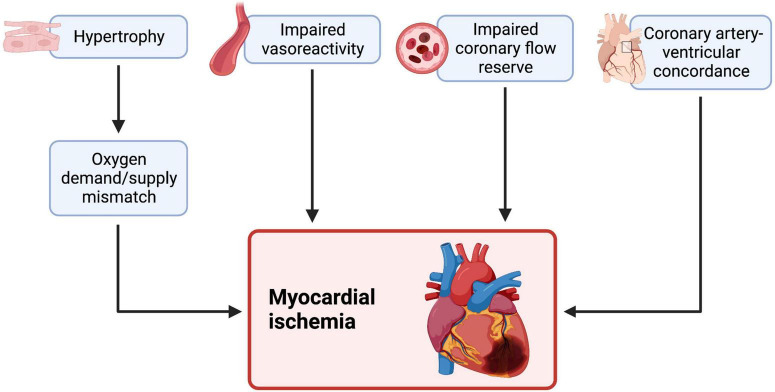
Myocardial ischemia and an impaired coronary flow reserve is independently associated with cardiac dysfunction and heart failure in systemic LV and sRV failure. sRV patients also show impaired vascular reactivity to adenosine (a vasodilator) during stress. A hypertrophied sRV may effectively outgrow its blood supply, creating an oxygen demand and supply mismatch and consequent ischemia. The RV is usually supplied throughout all phases of the cardiac cycle, but this may change in sRV, as some reports hypothesize that sRV perfusion is diastole-dependent, causing ischemia. Therefore, myocardial ischemia in sRV may be ischemia in sRV may be explained by various mechanisms, some of which may be amenable to treatment. We created the figure with biorender.com.

A variety of mechanisms underlie myocardial ischemia and perfusion defects in sRV patients (Figure 3). Vasoreactivity to adenosine is impaired in both d-TGA and cc-TGA patients (39, 44). sRV hypertrophy may also play a role in the development of myocardial ischemia by creating a mismatch between O_2_ demand and blood supply by the right coronary artery (3). Lastly, perfusion to the sRV is diastole-dependent, in contrast to the congenitally normal heart where the subpulmonic RV receives coronary blood flow throughout the cardiac cycle (45). However, whether sRV hypertrophy produces clinically measurable cardiac dysfunction is controversial. Grothoff et al. found positive septal movement due to disproportionate sRV hypertrophy supported sRV function without impairing LV function (46), whereas Hornung et al. reported a correlation between the degree of sRV hypertrophy and dysfunction of both ventricles (40). It is worth noting that both studies assessed patients with similar median post-operative intervals (21.5 and 24 years, respectively). In short, sRV hypertrophy may play a role in causing myocardial perfusion defects and consequent sRV dysfunction, although further studies are needed.

### 2.3. Tricuspid regurgitation

In sRV, the tricuspid valve becomes the systemic atrioventricular (SAVV) instead of the mitral valve, chronically causing tricuspid dysfunction. The tricuspid valve is ill-equipped to serve as the SAVV due to morphological differences between it and the mitral valve, predisposing it to TR (47). TR is a common complication in TGA patients and causes volume overload, leading to sRV dilatation, and eventual heart failure (48). Indeed, TR is a significant independent predictor of adverse cardiac events in d-TGA and cc-TGA patients (discussed below). Szymański et al. identified three distinct types of TR in sRV patients: annular dilatation, valvular prolapse, and valve tethering (48). Asymmetric tethering occurs in ventricular interdependence resulting in right-to-left septal shift and consequent TR (49). Compared to valvular prolapse and annular dilatation, sRV with tricuspid valve tethering had a more spherical RV, increased RV cavity area, lower RV fractional area change, and lower annular velocities in Doppler tissue imaging (48). These patients also had significantly greater BNP levels than patients with valve prolapse (48). These results indicate that TR due to valve tethering is associated with a considerably greater degree of sRV dysfunction than annular dilatation or valve prolapse.

Moderate and severe TR was found in 27.5 and 20% of d-TGA patients post-atrial switch repair (50–52), developing ∼12 years after the procedure (51). A risk prediction model was recently devised by Woudstra et al. to stratify patients according to their likelihood for event-free survival, which included age > 30 years, > 1 year after Mustard/Senning surgery, prior ventricular arrhythmia, right or left ventricular dysfunction, and severe TR (53). Severe TR significantly increased the risk of death in d-TGA patients (HR = 7.00, *p* < 0.001) (53). d-TGA patients suffer from functional TR, secondary to annular dilatation of the sRV, rather than from intrinsic morphologic abnormalities of the tricuspid valve (11). Therefore, management focuses on restoring sRV function—tricuspid valve replacement is not warranted. Interestingly, at least moderate post-operative TR after the atrial switch significantly predicted shorter overall survival and transplant-free survival, pointing to TR as a potential cause rather than a consequence of sRV dysfunction (54).

One of the unique features of cc-TGA distinguishing it from d-TGA is the intrinsically abnormal tricuspid valve in the former. Studies have found that 94% of patients with cc-TGA suffered from intrinsic tricuspid valve abnormalities, including Ebstein anomaly, dysplasia, straddling, stenosis, and overriding (55). Indeed, heart failure due to severe TR and sRV dysfunction is the most common presentation of adults diagnosed with cc-TGA (56). Around 30% of patients with simple cc-TGA and 60% of those with associated lesions develop heart failure by 45 years of age. TR is a key prognostic determinant of the overall survival of cc-TGA patients (11). For instance, long-term survival (20 years) was 47% for patients with at least moderate TR and 93% for those without TR (57). TR was also the only independent risk factor for death in the multivariate analysis (57). TR in cc-TGA is mainly due to intrinsic tricuspid valve abnormalities, although chronic sRV dilatation and dysfunction also play a role (55, 57, 58). Therefore, tricuspid valve replacement surgery is often indicated in these patients (discussed below).

In summary, SAVV regurgitation in sRV has been incorporated into several prognostic prediction models to stratify cc-TGA and d-TGA patients. However, considering the multifactorial pathophysiology of sRV failure, it is difficult to delineate the exact contribution of TR in this context.

### 2.4. Mechanical dyssynchrony and arrhythmias

Approximately 25% of adult CHD-related admissions are due to arrhythmias (59). Bradyarrhythmias, tachyarrhythmias, and SCD are not uncommon in sRV patients and are potentially fatal. The recent Broberg et al. study showed atrial arrhythmias and ventricular arrhythmias to correlate with the primary clinical outcome of death, transplantation, or MCS device requirement in d-TGA patients after the Mustard/Senning procedure (60). Ventricular arrhythmias were also part of a 5-year risk prediction model of the primary outcome (60). Indeed, d-TGA patients after atrial switch surgery are among the CHD patient groups at the highest risk of sudden cardiac death (SCD) (4, 61), with numerous potential triggers (62). On long-term follow-up (33.6 years), SCD is the most common cause of death in d-TGA post-atrial switch, especially in adolescents (63). The atrial switch surgery for d-TGA may damage the sinoatrial node/atrial conduction tissue or temporarily decrease blood flow to the sinoatrial node, causing post-operative sinus node dysfunction—estimated prevalence ≤ 60% at 20-year follow-up (51, 64). Intra-atrial re-entrant tachycardia (IART)—an atypical form of atrial flutter on ECG—is the most common type of supraventricular tachycardia after the atrial switch procedure, seen in 25% of patients on long-term follow-up (61). It likely arises because of fibrosis from the presence of suture lines and patches within the RA after surgery. IARTs must be recognized and treated promptly, as secondary ventricular tachycardias and fibrillation can develop because of increased atrioventricular conduction (64). Primary ventricular tachycardias frequently complicate sRV failure and cause SCD in d-TGA and cc-TGA patients (4, 62). Arrhythmias may also develop because of TR and secondary atrial dilatation. Atrial arrhythmias are also linked with SCD in d-TGA patients post-atrial switch (62).

Cardiac dyssynchrony is defined as non-synchronous peak myocardial fiber shortening caused by a delay in the electrical and mechanical activation of the heart (65). Mechanical dyssynchrony impairs efficient ventricular contraction, reducing cardiac output and contributing to heart failure progression. Cardiac dyssynchrony contributes to progressive heart failure and mortality in congenitally normal patients with left bundle-branch block (LBBB) (66–68). Right bundle-branch blocks (RBBBs) frequently develop after atrial-switch surgery for d-TGA, causing delayed activation-induced sRV electromechanical dyssynchrony and dysfunction (69–71). Reportedly, 10% of d-TGA patients, after the atrial switch procedure, develop conduction disturbances with significant QRS prolongation > 120 ms, a marker of electromechanical dyssynchrony (53). Electromechanical dyssynchrony induces sRV dysfunction in d-TGA patients and correlates with exercise intolerance and major adverse cardiac events, including heart failure (72). Broberg et al. recently showed QRS prolongation > 120 ms as an independent 5-year predictor of a composite primary outcome of death, transplantation, or MCS device implantation (60). Cardiac fibrosis may damage the conduction system, leading to dyssynchrony. Indeed, fibrosis is associated considerably with delayed septal shortening, lengthening, and prolonged sRV isovolumic contraction times in sRV patients post-atrial switch (73). Myocardial perfusion defects also induce ventricular dyssynchrony (73–75).

A key distinction between cc-TGA and d-TGA regarding clinical presentation is the predisposition for AV block in the former. AV conduction abnormalities are widespread in cc-TGA patients because of malalignment of the atrioventricular septum and a longer course taken by the conduction system to reach the ventricles (11, 76). A ventricular septal defect is the most commonly associated lesion with TGA, and its closure may precipitate an iatrogenic AV block (61, 77). Congenital conduction abnormalities with heart block can also occur but run a low mortality risk (78). Huhta et al. reviewed data on 107 cc-TGA patients and found that 23 patients (22%) had complete AV block and an annual risk of developing *de novo* AV block of approximately 2% (79). Complete AV block is the initial presentation of 10% of cc-TGA patients, while 20–30% present with second-degree AV block (80). In the absence of associated abnormalities, cc-TGA patients remain asymptomatic throughout early life and present later with progressive heart block, attributed to fibrosis-induced discontinuity of the abnormally long conduction system (76). Improvements in pharmacotherapies and invasive treatment for sRV patients have prolonged the life of individuals with an sRV, increasing the risk of chronic sRV dysfunction-related conduction abnormalities; the incidence of complete AV block in cc-TGA patients is 30–50% by 50 years of age (79–81). Lastly, as discussed above, TR affects many cc-TGA patients, requiring tricuspid valve surgery. However, AV conduction abnormalities are common after tricuspid valve surgery (82). Nederend et al. reported that 41% (9/22) of cc-TGA patients developed new-onset AV block after tricuspid valve replacement (83). Together, these findings demonstrate sRV dyssynchrony to be very prevalent in cc-TGA patients, secondary to a variety of etiologies, including fibrosis, perfusion defects, associated lesions, and tricuspid valve replacement.

Such conduction abnormalities are often treated with chronic subpulmonary LV (i.e., univentricular) pacing. However, pacing-induced dyssynchrony is a well-known phenomenon that worsens sRV dysfunction and contributes to sRV failure. Indeed, LV pacing is a risk factor for progressing sRV dysfunction in cc-TGA patients (84). For instance, patients receiving chronic subpulmonary pacing have a significantly longer QRS complex duration, higher NT-pro-BNP, and pronounced sRV systolic dysfunction than patients with no conduction abnormalities (83). sRV patients receiving chronic pacing post-tricuspid valve surgery are also at higher risk of mortality, ventricular assist device implantation, and requirement for cardiac resynchronization therapy (CRT) compared to patients with native AV conduction (83). Moore et al. retrospectively analyzed 106 adult CHD cases, including TGA. They found 25% of patients suffer from univentricular pacing-induced cardiomyopathy, with a higher risk of admission due to heart failure than those without (44% vs. 15%) (85). Notably, only individuals with a high pacing burden (when ventricular pacing exceeded 70%) developed pacing-induced cardiomyopathy develop; no patients with ventricular pacing < 70% developed pacing-induced ventricular dysfunction (85). Mechanistically, univentricular pacing may cause a septal shift toward the subpulmonary LV, with consequent annular dilatation of the sRV and TR, which, in turn, causes volume overload and exacerbates sRV dilatation (86), leading to a vicious cycle of progressive TR and sRV dysfunction. For these reasons, current recommendations suggest upgrading to CRT in patients receiving univentricular pacing or using primary CRT in patients being considered for pacing. Clinical studies investigating CRT vs. univentricular pacing as the primary treatment modality are required to validate these suggestions.

## 3. Management of the failing systemic right ventricle

### 3.1. Medical therapy

Evidence-based guidelines on pharmacologic treatment of sRV failure have not been established (87). Drugs available for systemic LV failure are usually used to treat patients with sRV failure, with largely disappointing results. For example, current data do not support using β-blockers, ACEis, ARBs, or aldosterone receptor antagonists in cc-TGA or d-TGA patients (19, 87–95). Dos et al. recently reported that 14 sRV patients treated with the aldosterone-receptor antagonist eplerenone showed reduced collagen turnover biomarkers, but whether this correlated with an improvement in sRV function could not be determined (87).

The first sRV failure patient cohort (*n* = 18) treated with sacubitril/valsartan, an angiotensin receptor/neprilysin inhibitor (ARNI), reported significant improvements in 6-min walking distance, cardiac function assessed by echocardiography, and quality of life (96). Larger scale studies with longer follow-up times are needed to appreciate the effect of ARNI use in sRV patients. Also, data on whether ARNI reduces cardiovascular mortality and heart failure-related hospitalizations in sRV patients is lacking. A recent single-center study recruited a cohort of 18 sRV patients prescribed ARNI for a median duration of 13 months and compared blood pressure, cardiopulmonary stress testing, imaging, and NYHA functional class at baseline vs. after treatment (97). A significant decrease in NYHA functional class was noted, but all other findings were statistically insignificant, including heart failure-related hospitalizations (97).

Sodium-glucose cotransporter 2 (SGLT2) inhibitors, such as empagliflozin and dapagliflozin, decrease cardiovascular mortality and hospitalizations related to heart failure with reduced LV ejection fraction (98). SGLT2 inhibitors also relieve subpulmonic RV pathology and reduce mortality in experimental models of PHTN (99), suggesting that they might be effective in treating pressure overload-induced sRV dysfunction. A recent case report by Egorova et al. was the first published report describing dapagliflozin use in a 28-year-old woman with cc-TGA and NYHA class II-III sRV failure who did not tolerate ARNI therapy (100). Dapagliflozin use significantly improved the patient’s subjective and objective functional status, similar in magnitude to the ARNI regimen (100). Mechanistic pathways by which SGLT2 inhibitors mitigate cardiac dysfunction may involve decreases in renin-angiotensin-aldosterone system activation and sympathetic outflow (101). These findings suggest that SGLT2 inhibitors may be beneficial in sRV failure management, but larger-scale clinical trials are needed to validate these findings. Lastly, the anti-fibrotic drug pirfenidone has been shown to moderately reduce myocardial fibrosis—assessed by ECV fraction—in HFpEF patients after a year of follow-up in the recent PIROUETTE trial. However, anti-fibrotic medications have not yet been used in sRV patients with heart failure.

### 3.2. Pacemakers and implantable cardioverter-defibrillator placement

Arrhythmia prevention strategies in sRV patients are not well defined. Indeed, the burden of implantable cardioverter-defibrillator (ICD) placement in TGA patients is considerably greater than that of the current population; TGA is only second to tetralogy of Fallot for ICD implantation for adult CHDs. As per the European Society of Cardiology (ESC) 2020 guidelines on the management of adult CHDs, ICD implantation may be considered for secondary prevention of SCD in the setting of severe sRV dysfunction (ejection fraction < 35%) in the presence of additional risk factors, such as NYHA functional class II/III, severe SAVV regurgitation, and wide QRS duration ≥ 140 ms (102). Adjunctive therapies with antiarrhythmics that slow down heart rate and AV nodal conduction—such as β-blockers and amiodarone—are also beneficial in restoring 1:1 conduction in IARTs and other supraventricular tachycardias (81). Nevertheless, identifying sRV patients at risk of SCD remains challenging. The benefit of ICD as primary prevention of SCD in sRV is also unclear. Furthermore, current guidelines for ICD implantation in CHD patients often lead to over-implantation in low-risk and under-implantation in high-risk groups (103). Ladouceur et al. developed a risk stratification score to identify high-risk sRV and inform ICD guidelines (104). The score assesses patients’ future risk according to age, history of heart failure, syncope, severe sRV dysfunction, and left ventricular outflow obstruction (104).

Pacing is often required for d-TGA post-atrial switch repair patients with bradycardia and cc-TGA patients with heart block (4). However, as discussed above, univentricular pacing can worsen long-term sRV function. Therefore, biventricular pacing/CRT should be initiated in such patients or as a primary measure to prevent pacing-induced dyssynchrony. CRT has recently been shown to also significantly improve cardiac function in adult CHDs featuring a systemic LV (105). Janousek et al. demonstrated that CRT for spontaneous conduction abnormalities or pacing-induced dyssynchrony significantly improved sRV function on echocardiography and NYHA functional class (106). Similarly, Hofferbath et al. upgraded 14 cc-TGA patients receiving univentricular LV pacing to biventricular CRT due to sRV dysfunction and observed significant improvements in cardiac function (107). For comparison, 11 primary CRT recipients for AV block did not develop sRV dysfunction on short-term follow-up (107). Lastly, Yeo et al. compared 22 cc-TGA patients receiving subpulmonary LV pacing to 7 primary CRT recipients (108). Patients receiving univentricular pacing were more likely to experience progressive sRV dysfunction, TR, and deterioration in NYHA class compared to the CRT group (108). However, the indications of CRT in sRV patients need to be precisely defined. Egbe et al. recently compared the benefit of CRT, medical therapy, and tricuspid valve replacement in preventing sRV dysfunction. Only valve replacement was significantly beneficial before the onset of sRV dysfunction (84). A multicenter study assessed CRT response in 36 TGA patients (20 cc-TGA and 16 d-TGA) and found less successful reverse sRV remodeling than systemic LV remodeling after CRT (109). Baruteau et al. suggested that regurgitation of the systemic tricuspid valve may be less amenable to CRT than the mitral valve (80), underscoring the importance of decision-making regarding optimal management on a case-by-case basis while considering the patient’s underlying anatomical abnormalities.

These findings indicate that early tricuspid valve replacement may prevent future sRV dysfunction and heart failure. An international multicenter study by Kharbanda et al. showed that CRT significantly reduced NYHA class and QRS duration in sRV patients on pre-existing univentricular pacing, but primary CRT had no significant benefit (110). Therefore, the best pacing strategy in sRV patients in terms of longevity, safety profile, and magnitude of improvement in sRV function remains yet to be defined; more comparative observational studies on univentricular pacing upgraded to CRT vs. primary CRT use are needed. Studies thus far have utilized echocardiography to document changes in sRV function after univentricular pacing or CRT. Using CMR to characterize the structural and functional changes in the heart after pacing would strengthen such studies. New pacing techniques, such as conduction system pacing and leadless pacing, are used in congenitally normal cardiac patients. However, experience in CHDs, including sRV, is limited (111).

### 3.3. Tricuspid valve replacement

Precipitants of sRV failure may require invasive treatment. Treatment for patients with mild or moderate TR aims to reduce ventricular afterload and prevent arrhythmias (112). Patients with severe TR may be considered for TV replacement; valve repair has shown inferior outcomes (higher recurrence rates) compared to replacement (113, 114). However, SAVV replacement is a high risk procedure in patients with severely impaired sRV function. Transcatheter edge-to-edge valve repair with clip implantation has been used to treat mitral and TR in congenitally normal hearts. Case reports and series implanting clips in the SAVV tricuspid position in d-TGA and cc-TGA have also reported good clinical and echocardiographic improvement (115). Albeit more extensive studies with longer follow-ups are needed for validation, this percutaneous-based clip implantation may be suitable for sRV patients at high surgical risk (116).

The major determinant of post-operative sRV function after SAVV replacement is preoperative sRV ejection fraction. At a follow-up of ∼8 years, 63% of cc-TGA patients with preoperative sRV EF ≥ 40% maintained sRV function, compared to only 10% with a preoperative sRV EF ≤ 40% (117). In agreement with these findings, Egbe et al. attributed a significant survival benefit to early SAVV replacement in cc-TGA performed before the onset of sRV dysfunction, whereas CRT and medical therapy yielded no statistically significant benefits (84). Likewise, immediate post-operative SAVV regurgitation after the atrial switch operation negatively affects the survival of d-TGA patients (54). The ESC 2020 guidelines revised their 2010 recommendations for the management of adult CHDs, upgrading the indication for SAVV replacement in cc-TGA patients with SAVV regurgitation and mildly impaired sRV function (EF ≥ 40%) from class IIa to I (102), underscoring the importance of early intervention.

### 3.4. Heart transplantation and mechanical circulatory support devices

Patients with decompensated sRV failure unresponsive to or unsuitable for CRT and refractory to medical therapy should be referred to a transplant center (112). However, a scarcity of donors coupled with adult CHD patients facing more prolonged waiting times on transplant lists increases the risk of major adverse events, such as heart failure-related hospitalizations, multi-organ failure, and death (118–120). sRV patients are also at risk of developing PHTN, which relates to worse prognostic outcomes and increases the perioperative risk of a heart transplant, precluding such patients from transplant eligibility (121, 122). Even after a transplant, short-term survival in adult CHD transplant recipients is still inferior compared to congenitally normal patients (10, 123–125). Paradoxically, however, CHD transplant recipients tend to fare better on long-term follow-up than congenitally normal patients (10, 123–125).

Ventricular assist devices (VADs) are increasingly used to manage advanced heart failure patients as either a bridge to transplant, bridge to candidacy, or destination therapy—indications are based on the INTERMACS profile (122). LVAD placement in advanced chronic heart failure cases prolongs survival and improves the quality of life. On a much smaller scale, VAD implantation has been beneficial in managing advanced sRV—even congenitally complex cases—in isolated case reports and small case series (126–132). Studies thus far have shown demonstrable reductions in pulmonary arterial pressure and pulmonary capillary wedge pressure after VAD implantation, rendering patients eligible for a transplant; further decreases in these parameters are noted after the transplant (133). Fernandez et al. recently demonstrated better hemodynamic and cardiac functional outcomes following VAD implantation and concomitant SAVV replacement (133). Larger single-center and multi-institutional data representative of a heterogenous sRV patient population are needed to corroborate these findings and inform the development of evidence-based guidelines for VAD implantation in sRV patients.

## 4. Conclusion

The guarded prognosis of sRV is due to the lack of evidence-based prognostic markers and treatment guidelines, particularly regarding pharmacologic management. We divided the mechanisms underpinning sRV dysfunction into myocardial fibrosis, ischemia/perfusion defects, arrhythmias and dyssynchrony, and TR. This classification encompasses the multifactorial nature of sRV dysfunction. Nevertheless, most data on the mediators of sRV dysfunction stem from studies on congenitally normal hearts. Mechanistic differences between sRV and LV failure need to be anticipated, exemplified by the poor efficacy of approved heart failure medications in relieving the radiologic and functional deterioration of sRV patients. Although the recent studies discussed above have significantly contributed to the scientific literature, the sRV patient population still seems to be an underrepresented community in clinical trials compared to heart failure in the systemic LV. Advancements in pediatric cardiology and surgery have ensured that 88% of children—including those with an sRV—reach adulthood, producing an aging sRV population with an annually increasing risk of cardiac complications, including heart failure (50, 134). An evidence-based consensus regarding the many avenues of managing sRV is still lacking. Future robust clinical trials based on the foundational clinical data presented above are needed to better delineate the clinical care of the failing sRV.

## Author contributions

BS, TA, AS, and DA-B: conceptualization. BS, TA, AS, SA, and AR: writing—original draft preparation. AS, SA, and DA-B: writing—review and editing. DA-B: supervision. All authors have read and agreed to the published version of the manuscript.
